# Tris[bis­(2-methyl­prop­yl)dithio­phosphinato]bis­muth(III)

**DOI:** 10.1107/S1600536810015618

**Published:** 2010-05-08

**Authors:** Hazoor A. Shad, M. Azad Malik, M. Nawaz Tahir, Zahid H. Chohan, Khalid H. Thebo, Madeleine Helliwell

**Affiliations:** a Department of Chemistry, Bahauddin Zakariya University, Multan 60800, Pakistan; b School of Chemistry and The Manchester Material Science Centre, The University of Manchester, Oxford Road, Manchester M13 9PL, England; c Department of Physics, University of Sargodha, Sargodha, Pakistan

## Abstract

The title compound, [Bi(C_8_H_18_PS_2_)_3_], contains a Bi^III^ cation surrounded by three bis­(2-methyl­prop­yl)dithio­phosphinate anions, leading to a distorted octa­hedral coordination for the heavy metal. The Bi—S and S—P bond lengths are in the ranges 2.7694 (18)–2.8391 (17) and 2.019 (2)–2.035 (2) Å, respectively. The crystal structure is consolidated by C—H⋯S hydrogen bonds. Intra­molecular C—H⋯π inter­actions also play a role in stabilizing the mol­ecules.

## Related literature

For applications of organodithio derivatives of phospho­rus compounds, see: Ebert *et al.* (1994 ▶). For a related structure, see: Lawton *et al.* (1974 ▶).
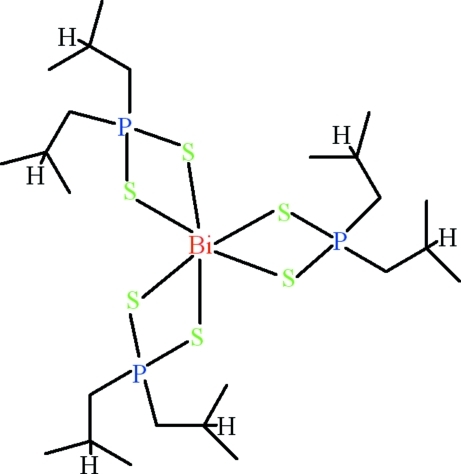


## Experimental

### 

#### Crystal data

[Bi(C_8_H_18_PS_2_)_3_]
*M* *_r_* = 836.92
Monoclinic, 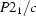

*a* = 13.954 (2) Å
*b* = 10.8719 (16) Å
*c* = 27.648 (3) Å
β = 119.312 (6)°
*V* = 3657.2 (9) Å^3^
*Z* = 4
Mo *K*α radiation
μ = 5.31 mm^−1^
*T* = 100 K
0.30 × 0.20 × 0.10 mm

#### Data collection

Bruker Kappa APEXII CCD diffractometer
Absorption correction: multi-scan (*SADABS*; Bruker, 2005 ▶) *T*_min_ = 0.293, *T*_max_ = 0.586
18769 measured reflections
6440 independent reflections
5175 reflections with *I* > 2σ(*I*)
*R*_int_ = 0.056

#### Refinement

*R*[*F* ^2^ > 2σ(*F* ^2^)] = 0.038
*wR*(*F* ^2^) = 0.072
*S* = 1.01
6440 reflections
319 parameters
H-atom parameters constrained
Δρ_max_ = 1.50 e Å^−3^
Δρ_min_ = −0.80 e Å^−3^

### 

Data collection: *APEX2* (Bruker, 2007 ▶); cell refinement: *SAINT* (Bruker, 2007 ▶); data reduction: *SAINT*; program(s) used to solve structure: *SHELXS97* (Sheldrick, 2008 ▶); program(s) used to refine structure: *SHELXL97* (Sheldrick, 2008 ▶); molecular graphics: *ORTEP-3 for Windows* (Farrugia, 1997 ▶) and *PLATON* (Spek, 2009 ▶); software used to prepare material for publication: *WinGX* (Farrugia, 1999 ▶) and *PLATON*.

## Supplementary Material

Crystal structure: contains datablocks global, I. DOI: 10.1107/S1600536810015618/wm2334sup1.cif

Structure factors: contains datablocks I. DOI: 10.1107/S1600536810015618/wm2334Isup2.hkl

Additional supplementary materials:  crystallographic information; 3D view; checkCIF report

## Figures and Tables

**Table 1:** Hydrogen-bond geometry (Å, °) *Cg*1, *Cg*2 and *Cg*3 are the centroids of the BI1/S1/P1/S2, BI1/S3/P2/S4 and BI1/S5/P3/S6 rings, respectively.

*D*—H⋯*A*	*D*—H	H⋯*A*	*D*⋯*A*	*D*—H⋯*A*
C11—H11*B*⋯S4	0.98	2.8200	3.624 (8)	139.00
C17—H17*A*⋯S6^i^	0.99	2.8200	3.806 (6)	173.00
C3—H3*C*⋯*Cg*1	0.98	2.97	3.665 (8)	129.00
C14—H14⋯*Cg*2	1.00	2.74	3.248 (8)	112.00
C22—H22⋯*Cg*3	1.00	2.90	3.345 (6)	108.00

Symmetry code: (i) 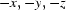.

**Fig. 1.**
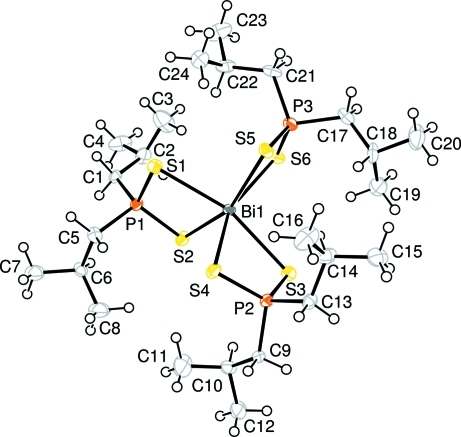
View of the molecular structure of (I) with the atom numbering scheme. Ellipsoids are drawn at the 50% probability level. H-atoms are shown by small circles of arbitrary radius.

**Fig. 2.**
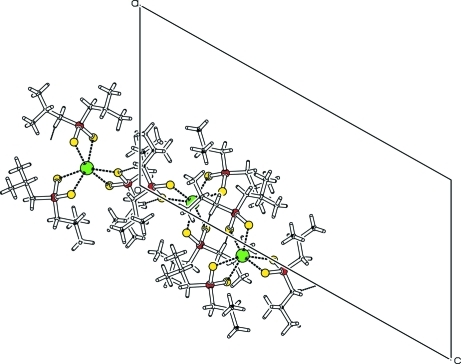
View of the partial packing of (I) which shows that molecules are interlinked through hydrogen bonding interactions.

## supplementary crystallographic information

### Comment

Organodithio-derivatives of phosphorus have several commercial applications, e.g. as additives to lubricant oils, petroleum additives, solvent extraction reagents for metals, flotation agents for mineral ores and as insecticides and pesticides in agriculture (Ebert *et al.*, 1994). We report here the crystal structure of the title compound, Bi(C_8_H_18_PS_2_)_3_, (I).

In the structure of (I) a Bi^III^ cation is present which is surrounded by three bis(2-methylpropyl)dithiophosphinate anions in a distorted octahedral coordination (Fig. 1). The Bi—S and S—P bond lengths are in the range of 2.7694 (18)—2.8391 (17) Å and 2.019 (2)—2.035 (2) Å, respectively. The S–Bi–S bond angles are in the range 72.90 (4)—163.04 (4)°. In comparison with (I), the crystal structure of bismuth(III) *O*,*O*^'^-di-isopropylphosphorodithioate (Lawton *et al.*, 1974), (II), shows a similar coordination behaviour of the central Bi^III^ cation. In structure (I) the four membered rings A (Bi1/S1/P1/S2), B (Bi1/S3/P2/S4) and C (Bi1/S5/P3/S6) are almost planar with r. m. s deviations from the plane of 0.066, 0.060 and 0.030 Å, respectively. The dihedral angles between A/B, A/C and B/C are 78.21 (3), 77.00 (4) and 75.78 (3)°, respectively. The molecules are stabilized due to hydrogen bonding interactions of the type C—H···S and additional C—H···π interactions (Table 1, Fig. 2).

### Experimental

To an aqueous solution (50 ml) of bismuth(III) chloride (1.57 g, 5 mmol) in a round bottom flask, an aqueous solution (50 ml) of sodium diisobutyldithiophosphinate (3.825 g, 15 mmol) was added drop-wise, leading to the formation of a precipitate within an hour. After the completion of the reaction, the precipitate was filtered off, washed with distilled water and re-crystallized in acetone. Colorless, clear and shiny prisms of compound (I) were collected from the mother liquor within 7 days.

### Refinement

The H-atoms were positioned geometrically (C–H = 0.98–1.00 Å) and refined as riding with *U*_iso_(H) = x*U*_eq_(C), where x = 1.5 for methyl and 1.2 for all other H-atoms.

### Crystal data

**Table:**

[Bi(C_8_H_18_PS_2_)_3_]	*F*(000) = 1688
*M**_r_* = 836.92	*D*_x_ = 1.520 Mg m^−^^3^
Monoclinic, *P*2_1_/*c*	Mo *K*α radiation, λ = 0.71073 Å
Hall symbol: -P 2ybc	Cell parameters from 2649 reflections
*a* = 13.954 (2) Å	θ = 2.0–26.4°
*b* = 10.8719 (16) Å	µ = 5.31 mm^−^^1^
*c* = 27.648 (3) Å	*T* = 100 K
β = 119.312 (6)°	Prisms, colorless
*V* = 3657.2 (9) Å^3^	0.30 × 0.20 × 0.10 mm
*Z* = 4	

### Data collection

**Table:**

Bruker Kappa APEXII CCD diffractometer	6440 independent reflections
Radiation source: fine-focus sealed tube	5175 reflections with *I* > 2σ(*I*)
graphite	*R*_int_ = 0.056
Detector resolution: 7.82 pixels mm^-1^	θ_max_ = 25.0°, θ_min_ = 2.1°
ω scans	*h* = −9→16
Absorption correction: multi-scan (*SADABS*; Bruker, 2005)	*k* = −12→12
*T*_min_ = 0.293, *T*_max_ = 0.586	*l* = −32→32
18769 measured reflections	

### Refinement

**Table:**

Refinement on *F*^2^	Primary atom site location: structure-invariant direct methods
Least-squares matrix: full	Secondary atom site location: difference Fourier map
*R*[*F*^2^ > 2σ(*F*^2^)] = 0.038	Hydrogen site location: inferred from neighbouring sites
*wR*(*F*^2^) = 0.072	H-atom parameters constrained
*S* = 1.01	*w* = 1/[σ^2^(*F*_o_^2^) + (0.0247*P*)^2^] where *P* = (*F*_o_^2^ + 2*F*_c_^2^)/3
6440 reflections	(Δ/σ)_max_ = 0.001
319 parameters	Δρ_max_ = 1.50 e Å^−^^3^
0 restraints	Δρ_min_ = −0.80 e Å^−^^3^

### Special details

**Table:**

Geometry. Bond distances, angles etc. have been calculated using the rounded fractional coordinates. All su's are estimated from the variances of the (full) variance-covariance matrix. The cell esds are taken into account in the estimation of distances, angles and torsion angles
Refinement. Refinement of *F*^2^ against ALL reflections. The weighted *R*-factor *wR* and goodness of fit *S* are based on *F*^2^, conventional *R*-factors *R* are based on *F*, with *F* set to zero for negative *F*^2^. The threshold expression of *F*^2^ > σ(*F*^2^) is used only for calculating *R*-factors(gt) *etc*. and is not relevant to the choice of reflections for refinement. *R*-factors based on *F*^2^ are statistically about twice as large as those based on *F*, and *R*- factors based on ALL data will be even larger.

### Fractional atomic coordinates and isotropic or equivalent isotropic displacement parameters (Å^2^)

**Table:**

	*x*	*y*	*z*	*U*_iso_*/*U*_eq_	
Bi1	0.06995 (2)	0.21172 (2)	0.17013 (1)	0.0163 (1)	
S1	−0.11857 (12)	0.33737 (13)	0.15017 (6)	0.0224 (4)	
S2	−0.03418 (12)	0.06325 (13)	0.21195 (6)	0.0227 (5)	
S3	0.26521 (12)	0.06889 (12)	0.22085 (6)	0.0206 (4)	
S4	0.22263 (12)	0.34033 (13)	0.26537 (6)	0.0226 (5)	
S5	0.09638 (12)	0.37496 (13)	0.09744 (6)	0.0211 (5)	
S6	−0.00195 (13)	0.08630 (13)	0.07039 (6)	0.0246 (5)	
P1	−0.14233 (12)	0.20298 (13)	0.19409 (6)	0.0178 (5)	
P2	0.33382 (11)	0.20960 (13)	0.27465 (6)	0.0177 (4)	
P3	0.02840 (13)	0.24188 (13)	0.03923 (6)	0.0201 (5)	
C1	−0.2816 (4)	0.1437 (5)	0.1584 (2)	0.0205 (17)	
C2	−0.3138 (5)	0.0524 (5)	0.1105 (2)	0.029 (2)	
C3	−0.3158 (6)	0.1108 (7)	0.0610 (3)	0.053 (3)	
C4	−0.4249 (5)	−0.0039 (6)	0.0953 (3)	0.042 (2)	
C5	−0.1299 (5)	0.2727 (5)	0.2559 (2)	0.0245 (19)	
C6	−0.1274 (5)	0.1858 (5)	0.3010 (2)	0.0220 (19)	
C7	−0.1946 (5)	0.2374 (6)	0.3260 (3)	0.039 (2)	
C8	−0.0108 (5)	0.1609 (7)	0.3458 (3)	0.044 (3)	
C9	0.4074 (4)	0.1487 (5)	0.3449 (2)	0.0191 (17)	
C10	0.3411 (5)	0.0739 (5)	0.3660 (2)	0.0247 (17)	
C11	0.2785 (6)	0.1557 (7)	0.3855 (3)	0.054 (3)	
C12	0.4183 (5)	−0.0086 (6)	0.4133 (3)	0.041 (2)	
C13	0.4438 (4)	0.2748 (5)	0.2656 (2)	0.0224 (17)	
C14	0.4075 (5)	0.3253 (5)	0.2072 (3)	0.030 (2)	
C15	0.4928 (5)	0.2942 (6)	0.1895 (3)	0.040 (2)	
C16	0.3886 (6)	0.4624 (6)	0.2045 (3)	0.049 (3)	
C17	0.1147 (5)	0.2134 (5)	0.0084 (2)	0.0269 (19)	
C18	0.2382 (5)	0.1969 (6)	0.0471 (3)	0.033 (2)	
C19	0.2663 (6)	0.0715 (6)	0.0754 (3)	0.047 (3)	
C20	0.2997 (7)	0.2164 (6)	0.0149 (3)	0.057 (3)	
C21	−0.1002 (5)	0.2894 (5)	−0.0203 (2)	0.0271 (19)	
C22	−0.1951 (5)	0.3198 (5)	−0.0093 (2)	0.0294 (19)	
C23	−0.3052 (6)	0.2852 (6)	−0.0595 (3)	0.051 (3)	
C24	−0.1956 (5)	0.4512 (5)	0.0067 (3)	0.037 (2)	
H1A	−0.29575	0.10347	0.18643	0.0243*	
H1B	−0.33205	0.21497	0.14372	0.0243*	
H2	−0.25795	−0.01508	0.12385	0.0354*	
H3A	−0.36686	0.18052	0.04853	0.0794*	
H3B	−0.33999	0.05021	0.03098	0.0794*	
H3C	−0.24193	0.13983	0.07090	0.0794*	
H4A	−0.48110	0.06063	0.08166	0.0634*	
H4B	−0.42123	−0.04269	0.12817	0.0634*	
H4C	−0.44382	−0.06596	0.06630	0.0634*	
H5A	−0.06166	0.32229	0.27309	0.0293*	
H5B	−0.19216	0.33033	0.24472	0.0293*	
H6	−0.16097	0.10564	0.28284	0.0263*	
H7A	−0.19284	0.17968	0.35365	0.0582*	
H7B	−0.27075	0.24944	0.29669	0.0582*	
H7C	−0.16348	0.31646	0.34387	0.0582*	
H8A	0.02384	0.23821	0.36437	0.0661*	
H8B	0.03078	0.12514	0.32915	0.0661*	
H8C	−0.01133	0.10330	0.37290	0.0661*	
H9A	0.46754	0.09564	0.34763	0.0227*	
H9B	0.44203	0.21868	0.37057	0.0227*	
H10	0.28712	0.02094	0.33501	0.0295*	
H11A	0.23623	0.10447	0.39746	0.0812*	
H11B	0.22833	0.20897	0.35489	0.0812*	
H11C	0.33058	0.20646	0.41655	0.0812*	
H12A	0.47201	0.04212	0.44395	0.0613*	
H12B	0.45684	−0.06368	0.40051	0.0613*	
H12C	0.37605	−0.05741	0.42616	0.0613*	
H13A	0.47870	0.34222	0.29272	0.0270*	
H13B	0.50028	0.21065	0.27415	0.0270*	
H14	0.33661	0.28482	0.18067	0.0356*	
H15A	0.46621	0.32181	0.15129	0.0597*	
H15B	0.50466	0.20502	0.19169	0.0597*	
H15C	0.56222	0.33569	0.21420	0.0597*	
H16A	0.45682	0.50375	0.23083	0.0730*	
H16B	0.33102	0.48069	0.21401	0.0730*	
H16C	0.36557	0.49180	0.16686	0.0730*	
H17A	0.08699	0.13844	−0.01474	0.0327*	
H17B	0.10462	0.28268	−0.01691	0.0327*	
H18	0.26297	0.26145	0.07661	0.0392*	
H19A	0.24302	0.00673	0.04726	0.0699*	
H19B	0.22812	0.06129	0.09694	0.0699*	
H19C	0.34582	0.06597	0.10025	0.0699*	
H20A	0.37893	0.21848	0.04074	0.0851*	
H20B	0.27669	0.29440	−0.00537	0.0851*	
H20C	0.28295	0.14866	−0.01148	0.0851*	
H21A	−0.08554	0.36287	−0.03677	0.0325*	
H21B	−0.12474	0.22296	−0.04830	0.0325*	
H22	−0.18579	0.26766	0.02257	0.0348*	
H23A	−0.36439	0.29763	−0.05054	0.0764*	
H23B	−0.30350	0.19859	−0.06890	0.0764*	
H23C	−0.31826	0.33705	−0.09109	0.0764*	
H24A	−0.12610	0.47020	0.04010	0.0546*	
H24B	−0.25678	0.46469	0.01400	0.0546*	
H24C	−0.20434	0.50492	−0.02371	0.0546*	

### Atomic displacement parameters (Å^2^)

**Table:**

	*U*^11^	*U*^22^	*U*^33^	*U*^12^	*U*^13^	*U*^23^
Bi1	0.0133 (1)	0.0208 (1)	0.0137 (1)	−0.0009 (1)	0.0056 (1)	0.0008 (1)
S1	0.0244 (8)	0.0201 (7)	0.0254 (8)	0.0014 (7)	0.0142 (7)	0.0049 (6)
S2	0.0204 (8)	0.0201 (8)	0.0286 (8)	0.0037 (7)	0.0128 (7)	0.0047 (6)
S3	0.0216 (8)	0.0189 (7)	0.0201 (8)	−0.0032 (6)	0.0092 (7)	−0.0030 (6)
S4	0.0175 (8)	0.0224 (8)	0.0240 (8)	0.0031 (7)	0.0071 (7)	−0.0025 (6)
S5	0.0228 (8)	0.0221 (8)	0.0185 (8)	−0.0033 (7)	0.0103 (7)	−0.0050 (6)
S6	0.0300 (9)	0.0225 (8)	0.0184 (8)	−0.0034 (7)	0.0095 (7)	0.0011 (6)
P1	0.0169 (8)	0.0174 (8)	0.0200 (8)	0.0023 (7)	0.0098 (6)	0.0035 (6)
P2	0.0149 (7)	0.0178 (8)	0.0186 (7)	0.0001 (7)	0.0068 (6)	−0.0005 (7)
P3	0.0230 (8)	0.0207 (8)	0.0163 (8)	0.0004 (7)	0.0093 (7)	−0.0002 (6)
C1	0.016 (3)	0.023 (3)	0.024 (3)	0.002 (3)	0.011 (3)	0.007 (3)
C2	0.021 (3)	0.031 (4)	0.031 (4)	−0.005 (3)	0.009 (3)	−0.008 (3)
C3	0.051 (5)	0.068 (5)	0.040 (4)	−0.026 (4)	0.023 (4)	−0.018 (4)
C4	0.031 (4)	0.037 (4)	0.042 (4)	−0.009 (3)	0.005 (3)	0.010 (3)
C5	0.036 (4)	0.015 (3)	0.030 (3)	0.002 (3)	0.022 (3)	0.005 (3)
C6	0.031 (4)	0.017 (3)	0.021 (3)	0.001 (3)	0.015 (3)	0.000 (2)
C7	0.036 (4)	0.047 (4)	0.031 (4)	0.006 (3)	0.015 (3)	0.013 (3)
C8	0.040 (4)	0.057 (5)	0.030 (4)	0.007 (4)	0.013 (3)	0.015 (3)
C9	0.016 (3)	0.023 (3)	0.016 (3)	0.002 (3)	0.006 (2)	0.000 (2)
C10	0.022 (3)	0.029 (3)	0.020 (3)	−0.004 (3)	0.008 (3)	0.000 (3)
C11	0.058 (5)	0.062 (5)	0.065 (5)	0.018 (4)	0.047 (5)	0.031 (4)
C12	0.044 (4)	0.051 (4)	0.039 (4)	0.014 (4)	0.029 (4)	0.018 (3)
C13	0.018 (3)	0.022 (3)	0.028 (3)	0.000 (3)	0.012 (3)	0.004 (3)
C14	0.019 (3)	0.035 (4)	0.031 (4)	−0.003 (3)	0.008 (3)	0.007 (3)
C15	0.035 (4)	0.050 (4)	0.037 (4)	−0.006 (4)	0.020 (3)	0.013 (3)
C16	0.038 (4)	0.037 (4)	0.071 (5)	0.001 (4)	0.026 (4)	0.023 (4)
C17	0.040 (4)	0.025 (3)	0.023 (3)	−0.004 (3)	0.021 (3)	−0.001 (3)
C18	0.032 (4)	0.036 (4)	0.045 (4)	0.005 (3)	0.030 (3)	−0.001 (3)
C19	0.044 (4)	0.058 (5)	0.046 (4)	0.015 (4)	0.028 (4)	0.019 (4)
C20	0.070 (6)	0.047 (5)	0.082 (6)	0.026 (5)	0.060 (5)	0.022 (4)
C21	0.035 (4)	0.026 (3)	0.012 (3)	−0.005 (3)	0.005 (3)	0.001 (3)
C22	0.023 (3)	0.030 (4)	0.028 (3)	−0.005 (3)	0.007 (3)	0.014 (3)
C23	0.038 (4)	0.037 (4)	0.051 (5)	0.001 (4)	0.001 (4)	0.005 (4)
C24	0.033 (4)	0.041 (4)	0.035 (4)	0.003 (3)	0.016 (3)	0.001 (3)

### Geometric parameters (Å, °)

**Table:**

Bi1—S1	2.7694 (18)	C5—H5A	0.9900
Bi1—S2	2.7747 (17)	C5—H5B	0.9900
Bi1—S3	2.8391 (17)	C6—H6	1.0000
Bi1—S4	2.8169 (16)	C7—H7A	0.9800
Bi1—S5	2.8373 (16)	C7—H7B	0.9800
Bi1—S6	2.7859 (15)	C7—H7C	0.9800
S1—P1	2.028 (2)	C8—H8A	0.9800
S2—P1	2.025 (2)	C8—H8B	0.9800
S3—P2	2.019 (2)	C8—H8C	0.9800
S4—P2	2.026 (2)	C9—H9A	0.9900
S5—P3	2.022 (2)	C9—H9B	0.9900
S6—P3	2.035 (2)	C10—H10	1.0000
P1—C1	1.813 (6)	C11—H11A	0.9800
P1—C5	1.799 (6)	C11—H11B	0.9800
P2—C9	1.820 (5)	C11—H11C	0.9800
P2—C13	1.816 (6)	C12—H12A	0.9800
P3—C17	1.810 (7)	C12—H12B	0.9800
P3—C21	1.818 (6)	C12—H12C	0.9800
C1—C2	1.536 (7)	C13—H13A	0.9900
C2—C3	1.497 (9)	C13—H13B	0.9900
C2—C4	1.522 (11)	C14—H14	1.0000
C5—C6	1.551 (8)	C15—H15A	0.9800
C6—C7	1.519 (10)	C15—H15B	0.9800
C6—C8	1.511 (10)	C15—H15C	0.9800
C9—C10	1.545 (9)	C16—H16A	0.9800
C10—C11	1.519 (11)	C16—H16B	0.9800
C10—C12	1.515 (9)	C16—H16C	0.9800
C13—C14	1.538 (9)	C17—H17A	0.9900
C14—C15	1.530 (11)	C17—H17B	0.9900
C14—C16	1.509 (9)	C18—H18	1.0000
C17—C18	1.530 (10)	C19—H19A	0.9800
C18—C19	1.525 (9)	C19—H19B	0.9800
C18—C20	1.524 (13)	C19—H19C	0.9800
C21—C22	1.533 (10)	C20—H20A	0.9800
C22—C23	1.530 (10)	C20—H20B	0.9800
C22—C24	1.497 (8)	C20—H20C	0.9800
C1—H1A	0.9900	C21—H21A	0.9900
C1—H1B	0.9900	C21—H21B	0.9900
C2—H2	1.0000	C22—H22	1.0000
C3—H3A	0.9800	C23—H23A	0.9800
C3—H3B	0.9800	C23—H23B	0.9800
C3—H3C	0.9800	C23—H23C	0.9800
C4—H4A	0.9800	C24—H24A	0.9800
C4—H4B	0.9800	C24—H24B	0.9800
C4—H4C	0.9800	C24—H24C	0.9800
			
Bi1···H14	3.6700	H7C···H5B	2.5700
S1···S2	3.349 (2)	H7C···H8A	2.5400
S1···C3	3.616 (8)	H8A···H5A	2.3800
S2···S1	3.349 (2)	H8A···H7C	2.5400
S3···S4	3.361 (2)	H8B···S2	2.9800
S4···C11	3.624 (8)	H8B···C11	3.0400
S4···S3	3.361 (2)	H8B···H5A	2.5900
S5···C24	3.684 (8)	H8B···H11A	2.5600
S5···S6	3.360 (2)	H8C···H7A	2.4600
S6···S5	3.360 (2)	H8C···S5^i^	3.0300
S6···C19	3.681 (10)	H9A···H12B	2.3200
S1···H3C	2.9500	H9B···H11C	2.4500
S2···H5A^i^	2.8800	H9B···C23^x^	3.0800
S2···H2	2.9900	H9B···H23A^x^	2.5100
S2···H8B	2.9800	H10···S3	3.0600
S3···H10	3.0600	H11A···C8	3.0800
S3···H14	2.9700	H11A···H8B	2.5600
S3···H5B^i^	3.1000	H11A···H12C	2.4500
S4···H16B	2.9600	H11B···S4	2.8200
S4···H1A^ii^	3.1100	H11C···H9B	2.4500
S4···H11B	2.8200	H11C···H12A	2.4900
S4···H6^ii^	3.1200	H11C···C20^iv^	3.0700
S4···H4B^ii^	3.1600	H12A···H11C	2.4900
S5···H14	3.1500	H12B···H9A	2.3200
S5···H8C^ii^	3.0300	H12B···H15A^xi^	2.5000
S5···H18	2.9300	H12C···H11A	2.4500
S5···H24A	2.9000	H13A···H16A	2.3700
S6···H19B	2.9300	H13B···H15B	2.3100
S6···H22	2.9800	H13B···H15C	2.6000
S6···H17A^iii^	2.8200	H13B···C16^xi^	3.0200
P1···H3C	3.0600	H13B···H16A^xi^	2.3500
P3···H19B	3.1300	H14···Bi1	3.6700
C3···S1	3.616 (8)	H14···S3	2.9700
C11···S4	3.624 (8)	H14···S5	3.1500
C19···S6	3.681 (10)	H14···H18	2.5500
C24···S5	3.684 (8)	H15A···H16C	2.4800
C1···H6	3.0300	H15A···H12B^v^	2.5000
C3···H22	3.0400	H15B···H13B	2.3100
C6···H1A	3.0100	H15C···H7B^xii^	2.5100
C8···H11A	3.0800	H15C···H13B	2.6000
C11···H20B^iv^	3.0800	H15C···H16A	2.5200
C11···H8B	3.0400	H16A···H13A	2.3700
C16···H13B^v^	3.0200	H16A···H15C	2.5200
C19···H23B^iii^	3.0000	H16A···H13B^v^	2.3500
C20···H11C^vi^	3.0700	H16B···S4	2.9600
C21···H7A^vi^	3.0900	H16C···H15A	2.4800
C23···H9B^vii^	3.0800	H17A···H19A	2.4600
C24···H20B^viii^	2.9800	H17A···S6^iii^	2.8200
H1A···C6	3.0100	H17B···H20B	2.2700
H1A···H4B	2.3300	H17B···H21A	2.5800
H1A···H6	2.3900	H18···S5	2.9300
H1A···S4^i^	3.1100	H18···H14	2.5500
H1B···H3A	2.4600	H19A···H17A	2.4600
H1B···H4A	2.5700	H19A···H20C	2.4900
H2···S2	2.9900	H19A···H23B^iii^	2.3600
H3A···H1B	2.4600	H19B···S6	2.9300
H3A···H4A	2.5500	H19B···P3	3.1300
H3B···H4C	2.4600	H19C···H20A	2.5300
H3B···H20C^iii^	2.4500	H20A···H4A^xii^	2.4200
H3C···S1	2.9500	H20A···H19C	2.5300
H3C···P1	3.0600	H20B···H17B	2.2700
H3C···H22	2.3100	H20B···C24^viii^	2.9800
H4A···H1B	2.5700	H20B···C11^vi^	3.0800
H4A···H3A	2.5500	H20C···H19A	2.4900
H4A···H20A^ix^	2.4200	H20C···H3B^iii^	2.4500
H4B···H1A	2.3300	H21A···H17B	2.5800
H4B···S4^i^	3.1600	H21A···H24C	2.4200
H4C···H3B	2.4600	H21B···H23B	2.2900
H5A···H8A	2.3800	H22···S6	2.9800
H5A···H8B	2.5900	H22···C3	3.0400
H5A···S2^ii^	2.8800	H22···H3C	2.3100
H5B···H7B	2.3600	H23A···H24B	2.4700
H5B···H7C	2.5700	H23A···H9B^vii^	2.5100
H5B···S3^ii^	3.1000	H23B···H21B	2.2900
H6···C1	3.0300	H23B···C19^iii^	3.0000
H6···H1A	2.3900	H23B···H19A^iii^	2.3600
H6···S4^i^	3.1200	H23C···H24C	2.5400
H7A···H8C	2.4600	H24A···S5	2.9000
H7A···C21^iv^	3.0900	H24B···H23A	2.4700
H7B···H5B	2.3600	H24C···H21A	2.4200
H7B···H15C^ix^	2.5100	H24C···H23C	2.5400
			
S1—Bi1—S2	74.33 (5)	C6—C7—H7B	110.00
S1—Bi1—S3	163.04 (5)	C6—C7—H7C	109.00
S1—Bi1—S4	98.71 (5)	H7A—C7—H7B	109.00
S1—Bi1—S5	89.46 (5)	H7A—C7—H7C	109.00
S1—Bi1—S6	101.39 (5)	H7B—C7—H7C	109.00
S2—Bi1—S3	92.74 (5)	C6—C8—H8A	109.00
S2—Bi1—S4	102.12 (5)	C6—C8—H8B	109.00
S2—Bi1—S5	159.27 (5)	C6—C8—H8C	109.00
S2—Bi1—S6	96.86 (5)	H8A—C8—H8B	109.00
S3—Bi1—S4	72.90 (4)	H8A—C8—H8C	110.00
S3—Bi1—S5	105.42 (5)	H8B—C8—H8C	109.00
S3—Bi1—S6	90.84 (5)	P2—C9—H9A	108.00
S4—Bi1—S5	92.90 (4)	P2—C9—H9B	108.00
S4—Bi1—S6	155.41 (6)	C10—C9—H9A	108.00
S5—Bi1—S6	73.37 (4)	C10—C9—H9B	108.00
Bi1—S1—P1	86.74 (7)	H9A—C9—H9B	107.00
Bi1—S2—P1	86.66 (7)	C9—C10—H10	109.00
Bi1—S3—P2	86.78 (7)	C11—C10—H10	109.00
Bi1—S4—P2	87.25 (6)	C12—C10—H10	109.00
Bi1—S5—P3	86.74 (7)	C10—C11—H11A	109.00
Bi1—S6—P3	87.91 (6)	C10—C11—H11B	109.00
S1—P1—S2	111.45 (11)	C10—C11—H11C	109.00
S1—P1—C1	112.09 (18)	H11A—C11—H11B	110.00
S1—P1—C5	107.3 (2)	H11A—C11—H11C	110.00
S2—P1—C1	109.78 (19)	H11B—C11—H11C	109.00
S2—P1—C5	111.8 (2)	C10—C12—H12A	109.00
C1—P1—C5	104.2 (3)	C10—C12—H12B	110.00
S3—P2—S4	112.39 (10)	C10—C12—H12C	110.00
S3—P2—C9	109.08 (19)	H12A—C12—H12B	109.00
S3—P2—C13	108.80 (19)	H12A—C12—H12C	109.00
S4—P2—C9	112.8 (2)	H12B—C12—H12C	110.00
S4—P2—C13	110.81 (19)	P2—C13—H13A	109.00
C9—P2—C13	102.5 (3)	P2—C13—H13B	109.00
S5—P3—S6	111.82 (9)	C14—C13—H13A	109.00
S5—P3—C17	109.6 (2)	C14—C13—H13B	108.00
S5—P3—C21	112.82 (19)	H13A—C13—H13B	108.00
S6—P3—C17	112.43 (19)	C13—C14—H14	108.00
S6—P3—C21	107.6 (2)	C15—C14—H14	108.00
C17—P3—C21	102.2 (3)	C16—C14—H14	108.00
P1—C1—C2	118.8 (5)	C14—C15—H15A	110.00
C1—C2—C3	112.4 (5)	C14—C15—H15B	109.00
C1—C2—C4	109.1 (6)	C14—C15—H15C	109.00
C3—C2—C4	111.0 (5)	H15A—C15—H15B	109.00
P1—C5—C6	117.4 (4)	H15A—C15—H15C	109.00
C5—C6—C7	111.4 (5)	H15B—C15—H15C	109.00
C5—C6—C8	111.1 (6)	C14—C16—H16A	109.00
C7—C6—C8	110.5 (5)	C14—C16—H16B	109.00
P2—C9—C10	117.7 (4)	C14—C16—H16C	109.00
C9—C10—C11	112.4 (5)	H16A—C16—H16B	109.00
C9—C10—C12	109.5 (6)	H16A—C16—H16C	109.00
C11—C10—C12	109.3 (5)	H16B—C16—H16C	109.00
P2—C13—C14	114.7 (4)	P3—C17—H17A	108.00
C13—C14—C15	110.4 (6)	P3—C17—H17B	108.00
C13—C14—C16	111.4 (5)	C18—C17—H17A	108.00
C15—C14—C16	110.3 (6)	C18—C17—H17B	108.00
P3—C17—C18	118.2 (4)	H17A—C17—H17B	107.00
C17—C18—C19	112.5 (6)	C17—C18—H18	108.00
C17—C18—C20	109.8 (6)	C19—C18—H18	108.00
C19—C18—C20	110.3 (6)	C20—C18—H18	108.00
P3—C21—C22	116.6 (4)	C18—C19—H19A	109.00
C21—C22—C23	110.4 (5)	C18—C19—H19B	109.00
C21—C22—C24	113.2 (6)	C18—C19—H19C	109.00
C23—C22—C24	110.8 (6)	H19A—C19—H19B	109.00
P1—C1—H1A	108.00	H19A—C19—H19C	110.00
P1—C1—H1B	108.00	H19B—C19—H19C	109.00
C2—C1—H1A	108.00	C18—C20—H20A	110.00
C2—C1—H1B	108.00	C18—C20—H20B	110.00
H1A—C1—H1B	107.00	C18—C20—H20C	109.00
C1—C2—H2	108.00	H20A—C20—H20B	110.00
C3—C2—H2	108.00	H20A—C20—H20C	109.00
C4—C2—H2	108.00	H20B—C20—H20C	109.00
C2—C3—H3A	110.00	P3—C21—H21A	108.00
C2—C3—H3B	109.00	P3—C21—H21B	108.00
C2—C3—H3C	110.00	C22—C21—H21A	108.00
H3A—C3—H3B	109.00	C22—C21—H21B	108.00
H3A—C3—H3C	109.00	H21A—C21—H21B	107.00
H3B—C3—H3C	109.00	C21—C22—H22	107.00
C2—C4—H4A	109.00	C23—C22—H22	107.00
C2—C4—H4B	109.00	C24—C22—H22	107.00
C2—C4—H4C	109.00	C22—C23—H23A	109.00
H4A—C4—H4B	109.00	C22—C23—H23B	109.00
H4A—C4—H4C	109.00	C22—C23—H23C	109.00
H4B—C4—H4C	109.00	H23A—C23—H23B	109.00
P1—C5—H5A	108.00	H23A—C23—H23C	110.00
P1—C5—H5B	108.00	H23B—C23—H23C	110.00
C6—C5—H5A	108.00	C22—C24—H24A	110.00
C6—C5—H5B	108.00	C22—C24—H24B	110.00
H5A—C5—H5B	107.00	C22—C24—H24C	109.00
C5—C6—H6	108.00	H24A—C24—H24B	110.00
C7—C6—H6	108.00	H24A—C24—H24C	109.00
C8—C6—H6	108.00	H24B—C24—H24C	109.00
C6—C7—H7A	109.00		
			
S2—Bi1—S1—P1	5.75 (6)	Bi1—S4—P2—C9	−131.4 (2)
S4—Bi1—S1—P1	−94.48 (7)	Bi1—S4—P2—C13	114.39 (18)
S5—Bi1—S1—P1	172.68 (6)	Bi1—S5—P3—S6	−3.68 (11)
S6—Bi1—S1—P1	99.77 (7)	Bi1—S5—P3—C17	−129.07 (19)
S1—Bi1—S2—P1	−5.77 (6)	Bi1—S5—P3—C21	117.7 (3)
S3—Bi1—S2—P1	163.10 (6)	Bi1—S6—P3—S5	3.75 (11)
S4—Bi1—S2—P1	90.01 (7)	Bi1—S6—P3—C17	127.6 (2)
S5—Bi1—S2—P1	−45.50 (15)	Bi1—S6—P3—C21	−120.7 (2)
S6—Bi1—S2—P1	−105.72 (7)	S1—P1—C1—C2	78.1 (4)
S2—Bi1—S3—P2	−107.07 (7)	S2—P1—C1—C2	−46.3 (4)
S4—Bi1—S3—P2	−5.23 (7)	C5—P1—C1—C2	−166.2 (4)
S5—Bi1—S3—P2	83.06 (7)	S1—P1—C5—C6	−171.1 (5)
S6—Bi1—S3—P2	156.02 (7)	S2—P1—C5—C6	−48.6 (6)
S1—Bi1—S4—P2	170.07 (7)	C1—P1—C5—C6	69.9 (6)
S2—Bi1—S4—P2	94.35 (7)	S3—P2—C9—C10	−59.2 (4)
S3—Bi1—S4—P2	5.21 (7)	S4—P2—C9—C10	66.4 (4)
S5—Bi1—S4—P2	−100.04 (7)	C13—P2—C9—C10	−174.4 (4)
S6—Bi1—S4—P2	−45.36 (13)	S3—P2—C13—C14	59.9 (4)
S1—Bi1—S5—P3	−99.46 (8)	S4—P2—C13—C14	−64.2 (4)
S2—Bi1—S5—P3	−61.48 (15)	C9—P2—C13—C14	175.3 (4)
S3—Bi1—S5—P3	88.79 (8)	S5—P3—C17—C18	47.6 (5)
S4—Bi1—S5—P3	161.85 (8)	S6—P3—C17—C18	−77.5 (5)
S6—Bi1—S5—P3	2.61 (8)	C21—P3—C17—C18	167.5 (4)
S1—Bi1—S6—P3	83.38 (8)	S5—P3—C21—C22	−63.2 (5)
S2—Bi1—S6—P3	158.71 (8)	S6—P3—C21—C22	60.6 (4)
S3—Bi1—S6—P3	−108.44 (8)	C17—P3—C21—C22	179.1 (4)
S4—Bi1—S6—P3	−60.85 (13)	P1—C1—C2—C3	−68.1 (7)
S5—Bi1—S6—P3	−2.59 (8)	P1—C1—C2—C4	168.4 (4)
Bi1—S1—P1—S2	−8.17 (9)	P1—C5—C6—C7	−139.3 (5)
Bi1—S1—P1—C1	−131.7 (2)	P1—C5—C6—C8	97.1 (6)
Bi1—S1—P1—C5	114.5 (2)	P2—C9—C10—C11	−80.6 (5)
Bi1—S2—P1—S1	8.16 (9)	P2—C9—C10—C12	157.8 (4)
Bi1—S2—P1—C1	132.95 (19)	P2—C13—C14—C15	−140.2 (5)
Bi1—S2—P1—C5	−111.9 (2)	P2—C13—C14—C16	96.9 (6)
Bi1—S3—P2—S4	7.52 (9)	P3—C17—C18—C19	75.4 (6)
Bi1—S3—P2—C9	133.4 (2)	P3—C17—C18—C20	−161.4 (4)
Bi1—S3—P2—C13	−115.58 (19)	P3—C21—C22—C23	−147.1 (4)
Bi1—S4—P2—S3	−7.58 (9)	P3—C21—C22—C24	88.1 (5)

Symmetry codes: (i) −*x*, *y*−1/2, −*z*+1/2; (ii) −*x*, *y*+1/2, −*z*+1/2; (iii) −*x*, −*y*, −*z*; (iv) *x*, −*y*+1/2, *z*+1/2; (v) −*x*+1, *y*+1/2, −*z*+1/2; (vi) *x*, −*y*+1/2, *z*−1/2; (vii) *x*−1, −*y*+1/2, *z*−1/2; (viii) −*x*, −*y*+1, −*z*; (ix) *x*−1, *y*, *z*; (x) *x*+1, −*y*+1/2, *z*+1/2; (xi) −*x*+1, *y*−1/2, −*z*+1/2; (xii) *x*+1, *y*, *z*.

### Hydrogen-bond geometry (Å, °)

**Table:**

Cg1, Cg2 and Cg3 are the centroids of the BI1/S1/P1/S2, BI1/S3/P2/S4 and BI1/S5/P3/S6 rings, respectively.

**Table:**

*D*—H···*A*	*D*—H	H···*A*	*D*···*A*	*D*—H···*A*
C11—H11B···S4	0.98	2.8200	3.624 (8)	139.00
C17—H17A···S6^iii^	0.99	2.8200	3.806 (6)	173.00
C3—H3C···Cg1	0.98	2.97	3.665 (8)	129.00
C14—H14···Cg2	1.00	2.74	3.248 (8)	112.00
C22—H22···Cg3	1.00	2.90	3.345 (6)	108.00

Symmetry codes: (iii) −*x*, −*y*, −*z*.
